# TCN 201 selectively blocks GluN2A-containing NMDARs in a GluN1 co-agonist dependent but non-competitive manner

**DOI:** 10.1016/j.neuropharm.2012.04.027

**Published:** 2012-09

**Authors:** S. Edman, S. McKay, L.J. MacDonald, M. Samadi, M.R. Livesey, G.E. Hardingham, D.J.A. Wyllie

**Affiliations:** a Centre for Integrative Physiology, University of Edinburgh, Hugh Robson Building, George Square, Edinburgh EH8 9XD, UK; b Institute of Neuroscience and Physiology, The Sahlgrenska Academy, University of Gothenburg, SE 405 30 Gothenburg, Sweden

**Keywords:** TCN 201, TCN 213, NMDA receptor, GluN2A-selective, Glycine, d-serine, Schild analysis

## Abstract

Antagonists that are sufficiently selective to preferentially block GluN2A-containing *N*-methyl-d-aspartate receptors (NMDARs) over GluN2B-containing NMDARs are few in number. In this study we describe a pharmacological characterization of 3-chloro-4-fluoro-*N*-[4-[[2-(phenylcarbonyl)hydrazino]carbonyl]benzyl]benzenesulphonamide (TCN 201), a sulphonamide derivative, that was recently identified from a high-throughput screen as a potential GluN2A-selective antagonist. Using two-electrode voltage-clamp (TEVC) recordings of NMDAR currents from *Xenopus laevis* oocytes expressing either GluN1/GluN2A or GluN1/GluN2B NMDARs we demonstrate the selective antagonism by TCN 201 of GluN2A-containing NMDARs. The degree of inhibition produced by TCN 201 is dependent on the concentration of the GluN1-site co-agonist, glycine (or d-serine), and is independent of the glutamate concentration. This GluN1 agonist-dependency is similar to that observed for a related GluN2A-selective antagonist, *N*-(cyclohexylmethyl)-2-[{5-[(phenylmethyl)amino]-1,3,4-thiadiazol-2-yl}thio]acetamide (TCN 213). Schild analysis of TCN 201 antagonism indicates that it acts in a non-competitive manner but its equilibrium constant at GluN1/GluN2A NMDARs indicates TCN 201 is around 30-times more potent than TCN 213. In cortical neurones TCN 201 shows only modest antagonism of NMDAR-mediated currents recorded from young (DIV 9–10) neurones where GluN2B expression predominates. In older cultures (DIV 15–18) or in cultures where GluN2A subunits have been over-expressed TCN 201 gives a strong block that is negatively correlated with the degree of block produced by the GluN2B-selective antagonist, ifenprodil. Nevertheless, while TCN 201 is a potent antagonist it must be borne in mind that its ability to block GluN2A-containing NMDARs is dependent on the GluN1-agonist concentration and is limited by its low solubility.

## Highlights

► TCN 201 is a potent and selective GluN1/GluN2A NMDAR antagonist. ► TCN 201 antagonism is dependent on the GluN1-agonist concentration. ► TCN 201 antagonism is independent on the GluN2-agonist concentration. ► TCN 201 blocks GluN2A-containing NMDARs in a non-competitive manner. ► TCN 201 allows pharmacological identification of native GluN2 A-containing NMDAR populations.

## 1 Introduction

NMDARs are a subtype of the ionotropic glutamate receptor family and are tetrameric assemblies, the majority of which contain two GluN1 subunits and two GluN2 subunits (Collingridge et al., 2009; Traynelis et al., 2010). It is thought that these subunits assemble in a ‘dimer of dimers’ configuration (Schorge and Colquhoun, 2003) forming a central non-selective cation-conducting ion channel pore that is permeable to Na^+^, K^+^ and Ca^2+^ ions but which is blocked by Mg^2+^ ions in a voltage-dependent manner (Traynelis et al., 2010). Alternative splicing of exons 5, 21 and 22 gives rise to eight GluN1 subunit isoforms (Sugihara et al., 1992) while 4 separate genes encode GluN2A-D subunits. These GluN2 subunits are spatially and temporally regulated (Monyer et al., 1994, 1992) and give rise to the majority of the distinct pharmacological and biophysical properties exhibited by NMDAR subtypes (Chen and Wyllie, 2006; Erreger et al., 2004; Traynelis et al., 2010). *In vivo* glutamate binds to the ligand-binding domain (LBD) of GluN2 subunits, while glycine (or d-serine) binds to the homologous structure in GluN1 subunits and activation of NMDARs has an absolute requirement that these sites are occupied by their respective ligands (Johnson and Ascher, 1987; Kleckner and Dingledine, 1988). Conversely, antagonism of NMDARs can be achieved by blocking agonist function at either GluN1 or GluN2 LBDs.

Relatively few NMDAR subtype-selective antagonists exist (Ogden and Traynelis, 2011) and until recently only the non-competitive allosteric inhibitors ifenprodil (Williams, 1993), CP 101,606 (Mott et al., 1998) and Ro 256981 (Fischer et al., 1997) are sufficiently selective for GluN2B-containing NMDARs to be identified unambiguously. For GluN2A-containing NMDARs the competitive antagonist NVP-AAM077 (Auberson et al., 2002) can be used to isolate GluN2A-containing NMDAR-mediated responses but care must be taken to use this antagonist at concentration that has minimal action at GluN2B-containing NMDARs since it only weakly discriminates between these two NMDAR subtypes (Frizelle et al., 2006; Neyton and Paoletti, 2006; Wyllie and Chen, 2007).

More promisingly, two compounds have recently been identified (Bettini et al., 2010), now referred to as TCN 201 (originally called Compound 1) and TCN 213 (originally Compound 13), that appear to be selective for GluN1/GluN2A over GluN1/GluN2B NMDARs. In a recent study we have characterized the nature of TCN 213 antagonism and have demonstrated that this compound displays a high selectivity for GluN2A-containing NMDARs and can be used to monitor, pharmacologically, the switch in NMDAR expression in developing cortical neurones (McKay et al., 2012). The mechanism of TCN 213 appears to be paradoxical in nature; whilst the compound selects for GluN2A-containing NMDARs, the potency of TCN 213 block is dependent on the concentration of glycine and not that of glutamate. Using Schild analysis, TCN 213 was found to possess an equilibrium constant (*K*_B_) of 2 μM. Although estimates of the concentration of glycine (or d-serine) *in vivo* have not been determined, unequivocally, they are likely to be in the micromolar range, while typically concentrations of at least 30 μM are contained in artificial cerebro-spinal fluid solutions when performing assays of NMDAR function to ensure saturation of the GluN1 binding site. Use of glycine (or d-serine) at this concentration, which is equal to 20 × its EC_50_ value at GluN1/GluN2A NMDARs (Chen et al., 2008), limits the effectiveness of TCN 213 due to this antagonist's comparatively low affinity. Therefore relatively high concentrations of TCN 213 need to be used to achieve substantial block of GluN2A NMDAR-mediated responses (McKay et al., 2012).

The present study reports the pharmacological characterization of TCN 201 – an antagonist suggested to be more potent than TCN 213 (Bettini et al., 2010) while still discriminating between GluN1/GluN2A and GluN1/GluN2B NMDARs. Our data show that TCN 201 is indeed more potent than TCN 213, but like TCN 213, its antagonism is also GluN1 co-agonist dependent. Furthermore the nature of its antagonism is not competitive and our results are consistent with it having an allosteric modulatory effect on glycine (or d-serine) binding. Complementary to our recent genetic approach to elucidate the GluN2 subunit dependency of NMDAR excitotoxicity (Martel et al., 2012), TCN 201 can also be used to assess the contribution of GluN2A subunits and adds to the list of new GluN2A-selective ligands in the pharmacological toolbox that can be used to elucidate NMDAR subunit composition and function.

## 2 Materials and methods

### 2.1 Plasmid constructs, cRNA synthesis and receptor expression in oocytes

Nomenclature of NMDA receptor subunits follows Collingridge et al. (2009) and Alexander et al. (2011). pSP64T-based plasmid constructs containing cDNA coding for rat GluN1-1a (i.e. the splice variant that lacks exon 5, but contains exons 21 and 22), hereafter termed GluN1 and wild-type rat GluN2A receptor subunits were prepared as described by (Chen et al., 2005). The rat GluN2B-containing cDNA expression vector was a gift by Stephen Traynelis (Emory University, Atlanta, GA). cRNA was synthesized as runoff transcripts as previously detailed (Chen et al., 2005, 2008; Erreger et al., 2007). Fluorescence intensity in ethidium bromide-stained agarose gels was employed to confirm the fidelity and yield of synthesized cRNAs. For recombinant receptor expression, GluN1 and GluN2 cRNAs were mixed at a nominal ratio of 1:1 and diluted with nuclease-free water to 5 ng μl^−1^.

Oocytes (Stage V–VI) were removed from *Xenopus laevis* that had been killed in accordance with current UK Home Office protocols and defolliculated by initial collagenase treatment, then manually using forceps. 23–37 nl of cRNA mix was injected into oocytes which were subsequently maintained in Barth's solution (composition in mM: NaCl 88, KCl 1, NaHCO_3_ 2.4, MgCl_2_ 0.82, CaCl_2_ 0.77, Tris-Cl 15, adjusted to pH 7.35 with NaOH and supplemented with 50 IU ml^−1^ penicillin, 50 μg ml^−1^ streptomycin, 50 μg ml^−1^ tetracycline) for 24–48 h at 19 °C to allow for receptor expression and then stored at 4 °C until required for electrophysiological measurements.

### 2.2 Culture of rat cortical neurones

Cortical neurones from E21 Sprague–Dawley rat embryos were cultured as described previously (Bading and Greenberg, 1991; Martel et al., 2009; Papadia et al., 2008) except that the Neurobasal-A growth medium contained B27 (Invitrogen, Paisley, UK), 1% rat serum (Harlan UK Ltd, Oxon, UK) and 1 mM glutamine. At days *in vitro* (DIV) 4, 1 ml growth medium containing 9.6 μM cytosine β-d-arabinofuranoside hydrochloride (AraC) was added to each well to inhibit glial cell proliferation. Culture media were replenished every 2 days after DIV 9 by replacing 1 ml of the conditioned media with 1 ml of fresh growth medium that lacked rat serum but was supplemented with glucose (10 mM).

### 2.3 Transfection of cortical neurones

Neurones were transfected on DIV 7 using Lipofectamine 2000 (Invitrogen) according to the manufacturer's suggested protocol. Briefly, pCis-GluN2A (0.4 μg) (Rutter and Stephenson, 2000) together with eGFP (0.2 μg) in a volume of 333 μl was added per well of a 24-well plate. After 5 h this transfection solution was removed and replaced with Neurobasal-A growth medium (2 ml per well). Transfected neurones were incubated to enable GluN2A expression for a further 2–3 days (DIV 9–10). We have previously demonstrated that co-transfection of β-globin and eGFP did not significantly alter ifenprodil sensitivity of DIV 7–11 neurones (McKay et al., 2012) and in the present study non-transfected DIV 9–10 cells were used as ‘controls’ in sister cultures. Transfection efficiency was approximately 5% with >99% of eGFP-expressing cells being identified as NeuN-positive while <1% were GFAP positive (Soriano et al., 2008). Electrophysiological recordings were made from transfected neurones 48–72 h post-transfection.

### 2.4 Electrophysiological recordings

Two-electrode voltage-clamp (TEVC) recordings were made using a GeneClamp 500 amplifier (Molecular Devices, Sunnyvale, CA) at room temperature (18–21 °C) from oocytes placed in a bath that was perfused with a solution comprising (in mM): NaCl 115, KCl 2.5, HEPES 10, BaCl_2_ 1.8, EDTA 0.01; pH 7.4 with NaOH. Note EDTA was included to chelate contaminating low nanomolar levels of Zn^2+^ that potently block of GluN2A-containing NMDARs in a voltage-independent manner. Current and voltage electrodes were made from thin-walled borosilicate glass (GC150TF-7.5, Harvard Apparatus, Kent, UK) using a PP-830 electrode puller (Narishige Instruments, Tokyo, Japan) and when filled with 0.3 M KCl possessed resistances of between 1 and 2 MΩ. TEVC recordings were performed at −30 or −40 mV. Currents were filtered at 10 Hz and digitized online at 100 Hz, *via* a Digidata 1200 A/D interface (Molecular Devices, Union City, CA, USA), using WinEDR 3.1.9 software (Strathclyde Electrophysiology Software, Strathclyde University, UK).

Whole-cell NMDA-evoked currents in rat cultured cortical neurones were recorded using an Axopatch 200B amplifier (Molecular Devices) using patch-pipettes made from thick-walled borosilicate glass with a tip resistance of 4–8 MΩ that were filled with an ‘internal’ solution that contained (in mM): K-gluconate 141, NaCl 2.5, HEPES 10, EGTA 11; pH 7.3 with KOH. Experiments were conducted at room temperature (18–21 °C) in an ‘external’ solution containing (in mM): NaCl 150, KCl 2.8, HEPES 10, CaCl_2_ 2, glucose 10, EDTA 0.01; pH to 7.3 with NaOH. Picrotoxin (50 μM) and tetrodotoxin (300 nM) were included to block GABA_A_ receptor-mediated responses and action potential driven excitatory/inhibitory postsynaptic events, respectively. Access resistances were monitored and recordings where this changed by >20% were discarded. Currents were filtered at 2 kHz and digitized online at 5 kHz *via* a BNC-2090A/PCI-6251 DAQ board interface (National Instruments, Austin, TX, USA) and analyzed using WinEDR 3.1.9 software (Dr John Dempster, University of Strathclyde, Glasgow, UK).

### 2.5 Assessment of antagonist potencies

The concentration of TCN 201 required to inhibit 50% (IC_50_) of agonist-evoked responses were determined by fitting data to the equation:

$$I=I_{{\left[B\right]}_{\infty }}+\left(\left(I_{{\left[B\right]}_{0}}-I_{{\left[B\right]}_{\infty }}\right)/\left(1+{\left(\left[B\right]/{\text{IC}}_{50}\right)}^{n_{\text{H}}}\right)\right),$$

where *I* is current response, $I_{{\left[B\right]}_{0}}$ is predicted maximum current in the absence of antagonist, $I_{{\left[B\right]}_{\infty }}$ is the predicted minimum current in the presence of an infinite concentration of antagonist,[*B*] is the concentration of the antagonist and *n*_H_ represents the Hill coefficient. To obtain an overall mean IC_50_ value, data points were normalised to the predicted maximum, pooled and refitted with the equation, with the maximum of each curve being constrained to asymptote to 1 but with the minimum fitted as a free parameter (Otton et al., 2011; Wrighton et al., 2008). In addition we fitted data points where both the maximum and minimum were constrained (to 1 and 0, respectively). The *F* ratio was calculated from the following equation:

$$F=\frac{\left({\text{SSR}}_{1}-{\text{SSR}}_{2}\right)/\left({\text{DF}}_{1}-{\text{DF}}_{2}\right)}{{\text{SSR}}_{2}/{\text{DF}}_{2}},$$

where SSR_1_ and SSR_2_ are the sum of squared residuals of the constrained and unconstrained fits respectively and DF_1_ and DF_2_ are the degrees of freedom associated with each of the two fits.

### 2.6 Schild analysis

TCN 201 antagonism at the GluN1/GluN2A NMDAR was examined by the Schild method (Arunlakshana and Schild, 1959; Wyllie and Chen, 2007). Dose-ratios (*r*) were defined as the ratio of the concentration of agonist, in the presence of a fixed concentration of antagonist, required to evoke the same response that was obtained in the absence of the antagonist. Dose-ratios from individual oocytes were determined at low agonist concentrations by constructing a partial concentration-response curve generated in the absence of antagonist and in the presence of a series of increasing antagonist concentrations. Due to the lack of sufficient perfusion lines, the dose-ratio estimate for the higher concentrations of TCN 201 (3 and 10 μM) was determined in a separate series of experiments from those carried out for the lower two concentrations (0.3 and 1 μM). Each series of two-point concentration-response curves were plotted on a log–log scale and the slope of the line used to fit the initial (antagonist-free) two-point concentration-response curve was used to fit the remaining curves. These parallel fits were used to calculate an overall mean *r* value for each antagonist concentration [*B*], which were then used to construct a Schild plot. On a log–log scale the gradient of the line used to fit such data is predicted by the Schild equation to be unity for a competitive antagonist. Thus, if the slope of a ‘free’ fit was not significantly different from 1, then the results were taken to be consistent with the Schild equation, and the data were refitted with the slope fixed at 1, i.e. they were refitted with the Schild equation,

$$r-1=\frac{\left[B\right]}{K_{\text{B}}},$$

in which the only free parameter is the intercept on the *x*-axis and which gives the equilibrium constant for antagonist binding, *K*_B_. For ‘allosteric’ antagonism at equilibrium, the Schild plot deviates from unity at higher concentrations of antagonist (Christopoulos and Kenakin, 2002). Such data can be fitted with the following modified equation:

$$r-1=\frac{\left[B\right]\left(1-\alpha \right)}{\alpha \left[B\right]+K_{\text{B}}^{\text{\#}}},$$

where *α* is the allosteric constant and *K*_B_^#^ is the estimated allosteric antagonist dissociation constant. Note that if *α* = 0, this equation simply reduces to the Schild equation. In addition, and unlike the Schild equation, *K*_B_^#^ does not give an *r* value equal to 2 when *K*_B_^#^ equals [*B*] but rather will give an *r* value < 2 for 0 < *α* < 1 and thus *K*_B_^#^ should not be interpreted in the same way as the *K*_B_ of a competitive antagonist.

### 2.7 Chemicals

Glutamate, glycine and d-serine were purchased from Sigma–Aldrich (Poole, UK). TCN 201, TCN 213, NMDA, ifenprodil, picrotoxin and tetrodotoxin were purchased from Tocris Bioscience (Bristol, UK). Stock solutions (3 mM) of TCN 201 were made by dissolving the antagonist in DMSO. Due to the limited solubility of TCN 201 the maximum concentration used in our experiments was 10 μM – at higher concentrations a noticeable precipitate was observed in the external recording solutions used in either the TEVC experiments or whole-cell patch-clamp experiments.

### 2.8 Statistical analysis

Results are presented as mean ± standard error of the mean and statistical comparison between datasets was assessed using either Student's *t-*test (paired where appropriate) or analysis of variance (ANOVA) tests to determine whether differences between mean values were significant (*p* < 0.05). Analysis of the fitting inhibition curves to unconstrained or constrained minima was assessed using an *F*-test. Microcal Origin v8.0 software was used for graphical presentation.

## 3 Results

### 3.1 TCN 201 demonstrates potent but GluN1 co-agonist concentration-dependent inhibition of GluN1/GluN2A NMDAR-mediated responses

**Fig. 1:**
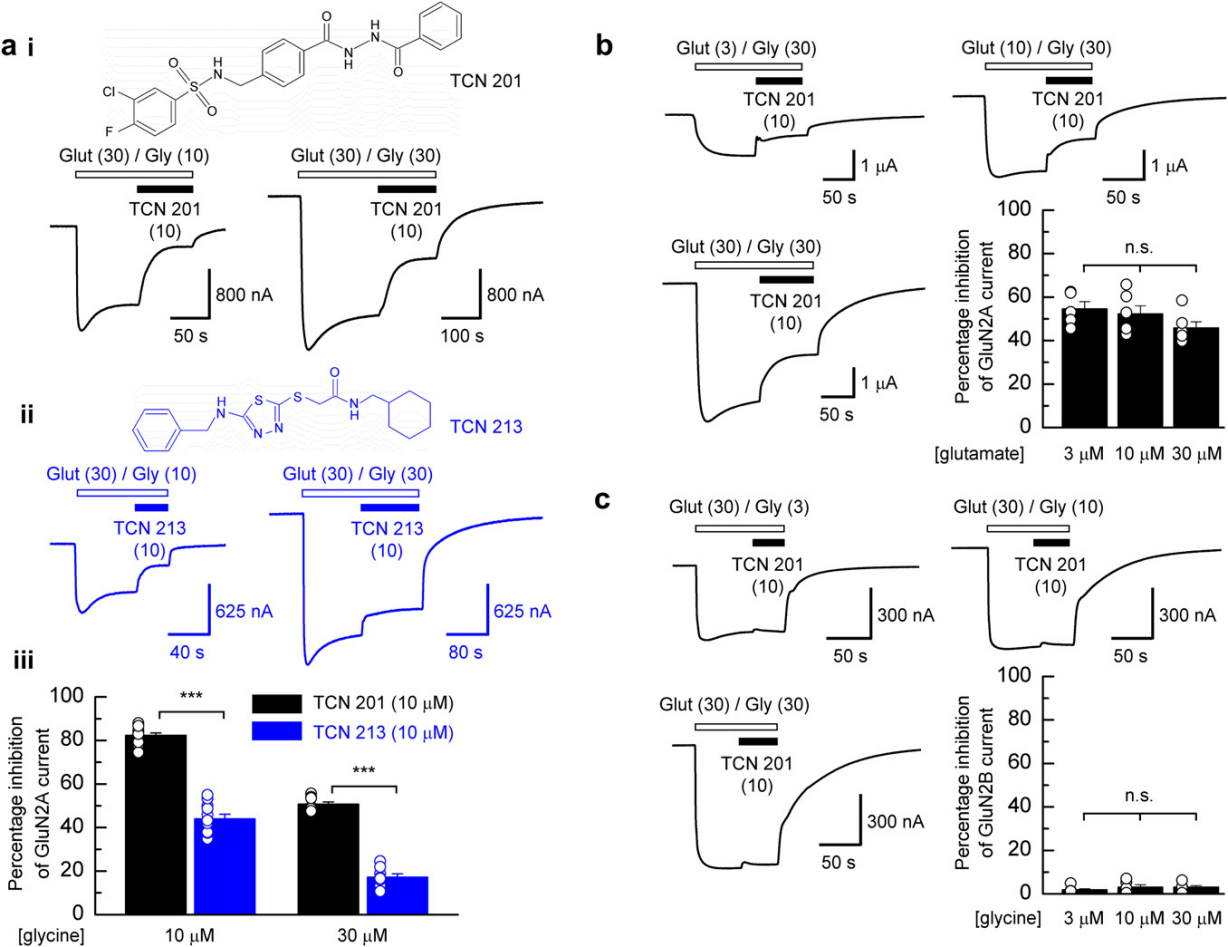
TCN 201 antagonism of NMDAR-mediated responses is both subtype- and glycine-dependent and more potent than TCN 213. (ai), upper panel, molecular structure of TCN 201. Lower panel, TEVC currents recorded from an oocyte expressing GluN1/GluN2A NMDARs in response to application of glutamate (30 μM) and glycine (10 μM, left-hand trace; 30 μM, righthand trace). TCN 201 (10 μM) was applied as indicated and inhibited the glutamate/glycine-evoked response but the extent of the inhibition was dependent on the glycine concentration. (aii), upper panel, molecular structure of TCN 213. Lower panel, a series of similar TEVC current traces in equivalent conditions, but recorded in the presence of TCN 213 (10 μM). (aiii), bar graphs summarizing the mean data obtained from a series of experiments that investigated the glycine-dependency of TCN 201 (10 μM, *n* = 12; 30 μM, *n* = 8) and TCN 213 (10 μM, *n* = 11; 30 μM, *n* = 9) antagonism of steady-state responses at GluN1/GluN2A NMDARs. (b), a series of representative TEVC current traces illustrating similar experiments as in (a), but where the glycine concentration was fixed (30 μM) and glutamate was applied at either 3, 10 or 30 μM. The bar graph summarizes the mean data obtained from a series of experiments that investigated the glutamate-dependency of TCN 201 antagonism of steady-state responses at GluN1/GluN2A and NMDARs at 3 μM (*n* = 5), 10 μM (*n* = 6) and 30 μM (*n* = 6). (c), a series of representative TEVC current traces illustrating similar experiments to that shown in (a), but for recordings made from oocytes expressing GluN1/GluN2B NMDARs. Note here the modest inhibition produced by TCN 201. The bar graph summarizes the mean data obtained from a series of experiments that investigated the glycine-dependency of TCN 201 antagonism of steady-state responses at GluN1/GluN2B NMDARs at 3 μM (*n* = 6), 10 μM (*n* = 6) and 30 μM (*n* = 6).

Fig. 1ai shows two representative TEVC current traces recorded from a *X. laevis* oocyte and mediated by recombinantly expressed GluN1/GluN2A NMDARs. The extent of the antagonism exhibited by TCN 201 is dependent on the glycine concentration present in the external recording solution and noticeably less inhibition is present in the righthand trace ([glycine] = 30 μM; 20 × its EC_50_ value). Fig. 1aii illustrates a comparable experiment where TCN 213, rather than TCN 201, was used – again note the decrease in the level of inhibition when glycine is present at the higher concentration. Fig. 1aiii shows the mean inhibition observed in a series of experiments for both TCN 201 and TCN 213 antagonism of GluN1/GluN2A NMDAR-mediated currents recorded in the presence of the lower (10 μM) or higher (30 μM) external glycine concentration. On average, and with glycine at 10 μM, TCN 201 produced 82.4 ± 1.1% (*n* = 12) inhibition whereas TCN 213 gave significantly less block (44.0 ± 2.2%; *n* = 11) of GluN1/GluN2A NMDAR-mediated currents (*p* < 0.001). Although both TCN 201 and TCN 213 gave less inhibition of currents evoked in the presence of the higher glycine concentration (50.7 ± 1.1%; *n* = 8 and 17.3 ± 1.4%; *n* = 9, respectively) the difference in potency between the two antagonists is still clearly evident (*p* < 0.001).

In contrast to the observed glycine dependency, the potency of block of TCN 201 (10 μM) was not dependent on the glutamate concentration used to evoke GluN1/GluN2A NMDAR-mediated currents (Fig. 1b). In these experiments glutamate was applied at either 3, 10 or 30 μM, while the glycine concentration was fixed at 30 μM. For each of the glutamate concentrations tested, the extent of block by TCN 201 was not significantly different (*F*_(2,14)_ = 1.839, *p* = 0.19, one-way ANOVA), with the mean values for the percentage inhibition being 54.5 ± 3.4% (*n* = 5; 3 μM), 52.2 ± 3.7% (*n* = 6; 10 μM) and 45.8 ± 2.8% (*n* = 6; 30 μM).

To assess TCN 201's selectivity for GluN1/GluN2A NMDARs a similar series of experiments to those illustrated in Fig. 1ai were performed but in oocytes that expressed GluN1/GluN2B NMDARs. As shown in Fig. 1c TCN 201 (10 μM) produced only slight inhibition of GluN1/GluN2B NMDAR-mediated currents. For glycine concentrations of 3, 10 and 30 μM the extent of block by TCN 201 was not significantly different (*F*_(2,15)_ = 0.8116, *p* = 0.46, one-way ANOVA), with the mean values for the percentage inhibition being 1.8 ± 0.6% (*n* = 6; 3 μM), 3.1 ± 1.0% (*n* = 6; 10 μM) and 3.1 ± 0.8% (*n* = 6; 30 μM). Thus our data demonstrate that TCN 201 selectively block GluN1/GluN2A NMDARs but in a manner that is dependent on the concentration of the GluN1-site agonist.

### 3.2 Inhibition curves for TCN 201 acting at GluN1/GluN2A NMDARs activated by glutamate and glycine or d-serine

**Fig. 2:**
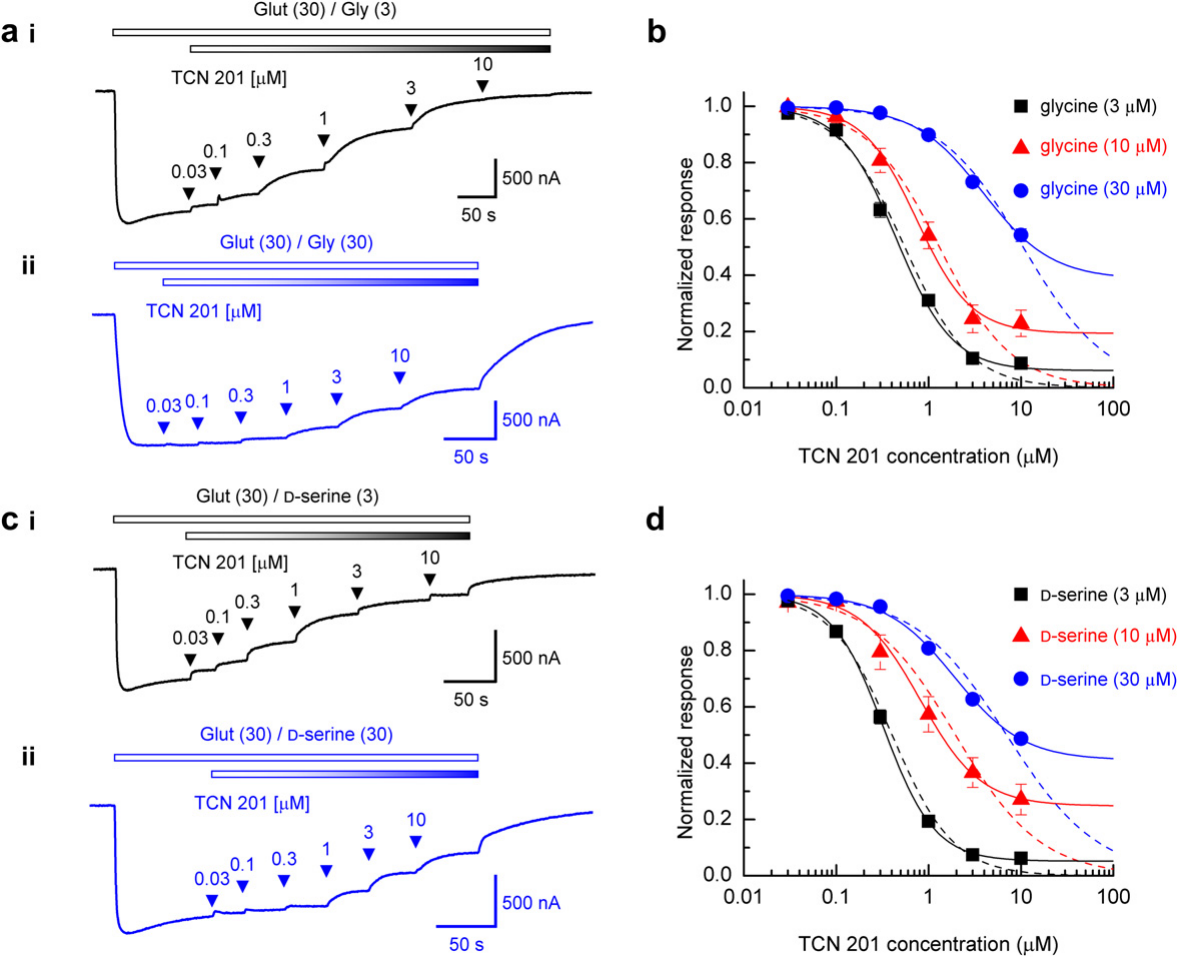
Inhibition curves for TCN 201 antagonism of GluN1/GluN2A NMDAR-mediated responses activated by co-agonists glycine or d-serine. (ai), TEVC trace recorded from an oocyte expressing GluN1/GluN2A NMDARs and voltage-clamped at −30 mV. The upper bar in this trace and in panels (aii), (ci) and (cii) indicates the duration of the bath application of glutamate/glycine, while the shaded bar in this panel (and in (ai), (ci) and (cii)) indicates the co-application TCN 201. Increasing concentrations of TCN 201 were applied, cumulatively, as indicated by the arrowheads. (aii), as in (ai), but currents are evoked using a higher concentration of glycine (30 μM). Note that TCN 201-mediated inhibition is less at this higher glycine concentration. (b), mean normalised inhibition curves for TCN 201 block of GluN1/GluN2A NMDAR-mediated currents evoked by glutamate (30 μM) and either 3 μM (*n* = 15; ■), 10 μM (*n* = 5; ) or 30 μM (*n* = 10; ) glycine. The solid curves show the fit with the minimum fitted as a free parameter, whereas the dashed curves show the fit of the data points when the minimum valued was constrained to 0 (see Materials and methods). (ci), as in (ai) but where currents were evoked by glutamate (30 μM) and d-serine (3 μM), again increasing concentrations of TCN 201 (0.03–10 μM) were applied, cumulatively, as indicated by the arrowheads. (cii), as in (ci), but where currents were the d-serine was 30 μM. (d), mean normalised inhibition curves for TCN 201 block of GluN1/GluN2A NMDAR-mediated currents activated by glutamate (30 μM) and either 3 μM (*n* = 6; ■), 10 μM (*n* = 6; ) or 30 μM (*n* = 6; ) d-serine.

We quantified the potency of TCN 201 by determining its IC_50_ value at GluN1/GluN2A NMDARs under conditions where either glycine or d-serine was used as the GluN1-site agonist. Additionally, since IC_50_ values are dependent on the concentration of the agonist used to evoke responses (Wyllie and Chen, 2007) three concentrations of each agonist were examined. As glycine and d-serine display the same potency at GluN1/GluN2A NMDARs (Chen et al., 2008) we were able to use the same concentrations of each agonist in this series of experiments. Fig. 2ai shows a representative TEVC current trace where the NMDAR-mediated current was evoked by a saturating concentration of glutamate (30 μM; (Erreger et al., 2007)) and glycine (3 μM; 2 × its EC_50_ value). Increasing concentrations (0.03–10 μM) of TCN 201 were applied cumulatively to inhibit the current. Fig. 2aii illustrates a similar experiment but under conditions where the glycine concentration was increased (30 μM; 20 × its EC_50_ value). It is evident that TCN 201 causes substantially less inhibition of the TEVC current trace illustrated in Fig. 2aii. The mean data obtained from a series of such experiments are shown in Fig. 2b. As described in Materials and Methods these data were fitted in two ways – either the predicted minimum value of the fit was left unconstrained (solid lines) or constrained to asymptote to zero (dashed lines). For each of the glycine concentrations examined fitting the data points with curves with an unconstrained minima led to significantly improved the fits (*F*_(4,3)_ = 10.34, *p* = 0.0422; 3 μM; *F*_(4,3)_ = 17.43, *p* = 0.0204; 10 μM; *F*_(4,3)_ = 36.28, *p* = 0.0071; 30 μM) which leads to the prediction that TCN 201 will not completely inhibit responses mediated by GluN1/GluN2A NMDARs. The calculated IC_50_ values (which in this context is the concentration of antagonist that gives 50% of the *maximal* amount of inhibition that can be achieved) and Hill slopes were 0.446 ± 0.026 μM, 1.42 ± 0.061 (*n* = 15, [glycine] = 3 μM), 0.746 ± 0.133 μM, 1.49 ± 0.21 (*n* = 5, [glycine] = 10 μM) and 3.89 ± 1.06 μM, 1.17 ± 0.11 (*n* = 10, [glycine] = 30 μM). We carried out a similar series of experiments and analyses using d-serine as the GluN1-site agonist (Fig. 2c, d) and again found that fitting the datasets with curves where the minima were left unconstrained produced significantly better fits than those where the minima were constrained to asymptote to zero (*F*_(4,3)_ = 106.95, *p* = 0.0014; 3 μM; *F*_(4,3)_ = 18.74, *p* = 0.0184; 10 μM; *F*_(4,3)_ = 38.63, *p* = 0.0065; 30 μM). The respective calculated IC_50_ values and Hill slopes obtained from these fits were 0.326 ± 0.018 μM, 1.57 ± 0.078 (*n* = 6, [d-serine] = 3 μM), 0.816 ± 0.231 μM, 1.33 ± 0.21 (*n* = 6, [d-serine] = 10 μM) and 1.92 ± 0.25 μM, 1.17 ± 0.07 (*n* = 6, [d-serine] = 30 μM).

Thus each of the datasets obtained at either the lower, intermediate or higher GluN1-site agonist concentration were better fitted with curves in which their minima were unconstrained. This indicates that TCN 201 will not produce complete block of NMDAR-mediated responses and is consistent with the notion that the antagonism is non-competitive in nature. Furthermore, the mean values for TCN 201 inhibition of GluN1/GluN2A NMDAR-mediated currents were significantly different (*p* = 0.0119, two-tailed *t*-test) when d-serine, rather than glycine was used as the GluN1-site agonist at the lowest concentration examined (3 μM). This ‘agonist-dependency’ of IC_50_ values is not unexpected given that IC_50_ values are dependent on agonist affinity and efficacy. Thus, we therefore decided to investigate further the nature of TCN 201's antagonism by carrying out Schild analysis which does not suffer from such limitations (Wyllie and Chen, 2007).

### 3.3 Schild analysis of TCN 201 antagonism of GluN1/GluN2A NMDAR-mediated currents

**Fig. 3:**
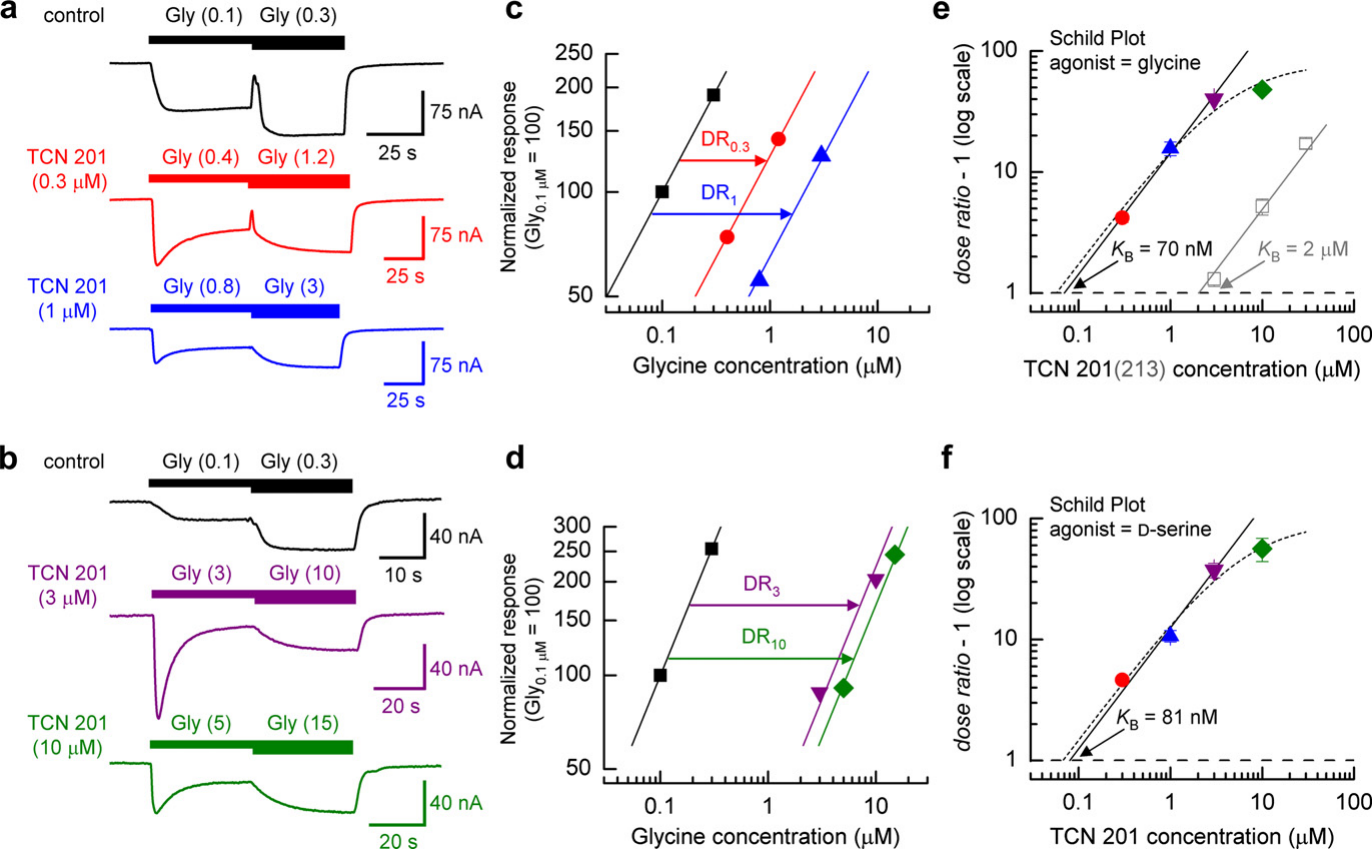
Schild analysis of TCN 201 antagonism of GluN1/GluN2A NMDAR-mediated responses. (a), illustration of a set of TEVC current traces, obtained from an oocyte expressing GluN1/GluN2A NMDARs, used to generate ‘two-point’ dose–response curves in either the absence or presence of TCN 201 (0.3 and 1 μM; *n* = 7). (b), as in (a) but for higher TCN 201 concentrations (3 and 10 μM; *n* = 7). (c) and (d), partial, low-concentration, dose–response curves obtained from the TEVC current traces illustrated in (a) and (b), respectively, and used to estimate dose ratios (DR_0.3, 1, 3, 10_). The slope of the fitted line to the control responses (no TCN 201; ■) was used to fit the responses obtained in the presence of 0.3 μM (), 1 μM (), 3 μM () and 10 μM (). (e), Schild plot for antagonism by TCN 201 of GluN1/GluN2A NMDARs using dose-ratios estimated from a series experiments such as that illustrated in (c) and (d). A ‘free’ fit of the 0.3, 1 and 3 μM TCN 201 data points gave has a slope of 0.98 which was considered not to be significantly different from 1 (95% confidence interval: 0.85–1.14). Thus the *solid line* is the fit of the respective data points to the Schild equation (i.e. the slope of this line is unity). The intercept on the *abscissa* (where the log_10_ value of the dose-ratio – 1 equals zero) gives an equilibrium constant (*K*_B_) value for TCN 201 of 70 nM. Data from McKay et al. (2012) where the *K*_B_ value for TCN 213 was determined, is illustrated in *grey* for reference. The dotted line shows the fit of all data points with a modified equation (see Material and methods; Christopoulos and Kenakin, 2002) that takes into account allosteric modulation of glycine binding by TCN 201. The fit predicts an allosteric *K*_B_^#^ value of 56 nM and an allosteric constant (*α*) of 0.0123. (f), Schild plot for antagonism by TCN 201 of GluN1/GluN2A NMDARs but using d-serine, rather than glycine, as the GluN1-site agonist. Again the *solid line* is the fit of the data to the Schild equation and gives a *K*_B_ value for TCN 201 in these experiments of 81 nM. The dotted line shows the fit of all data points to the modified equation and predicts an allosteric *K*_B_^#^ value of 66 nM and an allosteric constant of 0.0106.

Schild analysis (Arunlakshana and Schild, 1959; Wyllie and Chen, 2007) allows the unambiguous determination of the equilibrium constant (*K*_B_) for a competitive antagonist and is independent of the nature and concentration of agonist. Fig. 3a, b shows TEVC current traces that exemplify experiments carried out to determine the dose ratio (*r*) for shifts in glycine potency in the presence of increasing (0.3–10 μM) concentrations of TCN 201. Specifically, Fig. 3a depicts a series of recordings from a single oocyte where two concentrations of glycine (in the presence of glutamate (30 μM)) are applied in the absence (upper trace) or presence (middle and lower traces) of TCN 201 (0.3 and 1 μM). Fig. 3b shows a similar experiment but when the TCN 201 concentration was increased (3 and 10 μM). From these we constructed two-point concentration-response curves (Fig. 3c, d) and estimated *r* for each concentration of antagonist (*n* = 7). The mean *r* values obtained were 5.19 ± 0.4, 16.7 ± 2.1, 41.1 ± 1.5 and 49.0 ± 2.0 for 0.3, 1, 3 and 10 μM TCN 201, respectively. Note that the shift in the concentration-response curve in the presence of the highest concentration of TCN 201 (10 μM) is considerably less than would be predicted for competitive antagonism. Indeed this smaller than predicted shift is clearly observed in the Schild plot (Fig. 3e). However the first three data points (0.3, 1 and 3 μM TCN 201) appear to give a straight line. A linear regression fit of these data gives a line with a slope of 0.98 (95% confidence interval: 0.85–1.14). The fit of the data (solid line) illustrated in Fig. 3e shows a line whose slope has been constrained to unity and allows the derivation of the *K*_B_ for TCN 201 (70 nM). For comparison, data obtained from our study of TCN 213 antagonism (McKay et al., 2012) is illustrated – note the lower potency of this antagonist (*K*_B_ = 2 μM). Clearly however, the *entire* dataset for TCN 201 antagonism is not satisfactorily described by the Schild equation and this can be taken to indicate that TCN 201 is not acting in a competitive manner at the GluN1 agonist binding site (as indeed is also suggested by the data presented in Fig. 2). Thus, the dotted line (Fig. 3e) shows the complete dataset fitted with a modified equation (Christopoulos and Kenakin, 2002) which has previously been used to described agonist-surmountable ‘allosteric’ non-competitive antagonism at G-protein coupled receptors. This fit better describes the data and gives an allosteric *K*_B_^#^ of 56 nM and an allosteric constant (*α*) of 0.0123. This indicates that the maximum shift in the dose ratio that can be obtained with TCN 201 is around 80-fold (= 1/α). However such a shift would only be achieved with a TCN 201 concentration of approximately 275 μM which is well above its limit of solubility. In addition we carried out Schild analysis using d-serine, rather than glycine, as the GluN1-site agonist (Fig. 3f). A linear regression fit of the data points obtained with 0.3, 1 and 3 μM TCN 201 gives a line with a slope of 0.87 (95% confidence interval: 0.71–1.03). The fit of the data (solid line) illustrated in Fig. 3e shows a line whose slope has been constrained to unity and gives a *K*_B_ for TCN 201 of 81 nM – comparable to the value obtained when glycine was used as the GluN1-site agonist. Similar to our findings when glycine was used as the GluN1-site agonist, the data point at the highest concentration of TCN 201 (10 μM) gives an *r* value that deviates considerably from the linearity. Again using the modified equation we estimated an allosteric *K*_B_^#^ of 66 nM and an allosteric constant of 0.0106. Given the good agreement between these values with those obtained when glycine was used as the GluN1-site agonist and data reported in a recent study which has also examined the mechanism of action of TCN 201 (Hansen et al., 2012) it would seem unlikely that the deviation from linearity of the Schild plot arises from the limitation of TCN 201's solubility.

### 3.4 Antagonism by TCN 201 of NMDAR-mediated responses in rat cortical neurones

In a final series of experiments we assessed the ability of TCN 201 to antagonize NMDAR-mediated currents in rat cortical neurones to assess the utility of this compound in identifying NMDAR subunit populations. It should be noted that as we used NMDA (rather than glutamate) in these experiments we determined whether TCN 201 (10 μM) blocked, to the same extent, GluN1/GluN2A NMDAR-mediated currents elicited by NMDA/glycine compared with currents elicited by glutamate/glycine. We observed significantly less inhibition of NMDA/glycine-evoked currents (69.9 ± 4.1%; *n* = 12, *p* < 0.001, data not shown) compared to glutamate/glycine-evoked currents (91.2 ± 1.1%; Fig. 2b). This emphasizes the importance of considering the nature of the agonist used to evoke responses but establishes the maximum inhibition expected for TCN 201 block of NMDA/glycine-evoked currents for a population of NMDARs comprised *solely* of GluN1 and GluN2A NMDAR subunits.

**Fig. 4:**
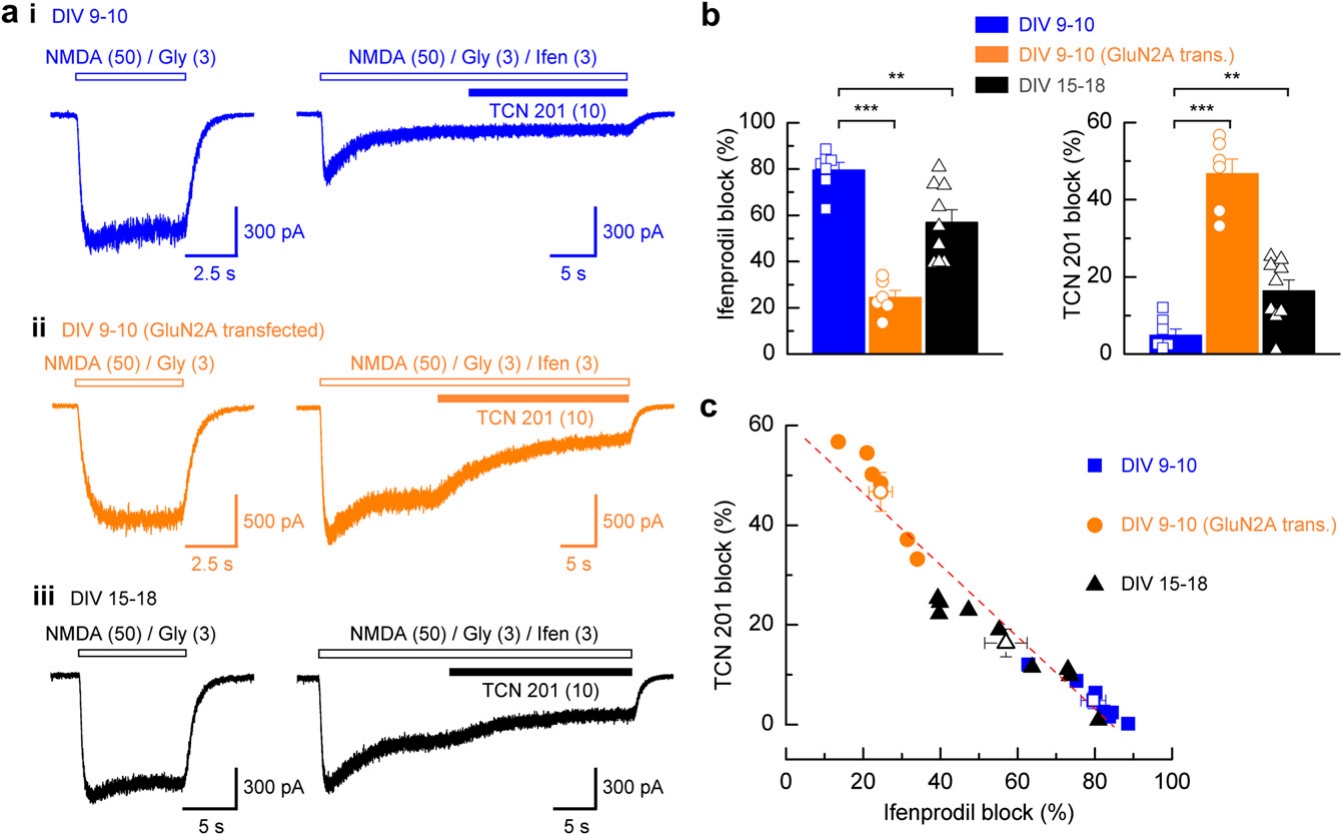
Antagonism by TCN 201 of native NMDAR-mediated responses in rat cortical cultures. (a), left, example steady-state whole-cell currents activated by NMDA (50 μM) and glycine (3 μM) recorded from cortical pyramidal cells voltage-clamped at −70 mV from (ai), DIV 9–10 neurones, (aii), DIV 9–10 neurones transfected with GluN2A NMDAR subunits, and (aiii), DIV 15–18 neurones. To the right, traces illustrate the sensitivity of each of these NMDAR-mediated currents to the GluN2B-selective antagonist, ifenprodil (3 μM) and the subsequent sensitivity of the ifenprodil-insensitive component of this current to TCN 201 (10 μM). (b), left, bar graph summarizing the mean percentage ifenprodil block of NMDAR-mediated currents recorded from DIV 9–10 neurones (*n* = 7), GluN2A-transfected DIV 9–10 neurones (*n* = 6), and DIV 15–18 neurones (*n* = 9). Right, mean percentage TCN 201 block (expressed as a percentage of the original current magnitude) of NMDAR-mediated currents recorded from neurones in each of the three categories illustrated in (a). (c), plot illustrating the extent of ifenprodil and TCN 201 antagonism of NMDA-evoked currents from the same cell. Despite a wide range in the amount of block produced by either ifenprodil or TCN 201 (particularly for recordings from GluN2A-transfected and from neurones in older cultures) the data show a strong (negative) correlation (*R*^2^ = 0.91).

Fig. 4a shows a series of NMDAR-mediated currents recorded from rat cortical neurones under three separate conditions. Fig. 4ai shows a representative recording from a neurone (DIV 9–10) where the predominantly expressed GluN2 subunit is anticipated to be GluN2B. As is illustrated in the righthand trace and quantified in Fig. 4b, ifenprodil (3 μM) produces substantial block (80 ± 3%, *n* = 7) of this current – as would be anticipated for a NMDAR population that contains a high proportion of GluN1/GluN2B NMDARs. Importantly, TCN 201 gives only a modest block of this current (5 ± 2%) which is comparable to the extent of TCN 201 block seen at recombinant GluN1/GluN2B NMDARs (Fig. 1c). To increase the number of GluN2A-containing NMDARs we transfected neurones with pCis-GluN2A plasmids (Rutter and Stephenson, 2000). NMDAR-mediated currents recorded from these neurones (Fig. 4aii) now displayed a reduced sensitivity to ifenprodil (24 ± 3% block, *n* = 6; Fig. 4b) but increased sensitivity to TCN 201 (47 ± 4% block, *n* = 6; Fig. 4b). Finally, the developmental increase in GluN2A expression was confirmed by recording NMDAR-mediated currents from neurones that had been cultured for longer time periods (Fig. 4aiii). In these neurones we observed that currents were less sensitive to ifenprodil (57 ± 5% block, *n* = 9; Fig. 4b) compared to those from DIV 9–10 cultures. This reduction in ifenprodil sensitivity was accompanied by an increase in the extent of the block produced by TCN 201 compared to that observed for currents in DIV 9–10 (non-transfected) cultures (16 ± 3% block, *n* = 9; Fig. 4b, *p* < 0.01). Furthermore, while the range of ifenprodil and TCN 201 block of currents showed considerable variation within each of these groups there was a strong inverse correlation (*R*^2^ = 0.91) in the sensitivities of currents to these two NMDAR subtype-selective antagonists (Fig. 4c). A similar observation has been documented for ifenprodil and TCN 213 block of NMDAR-mediated currents in cortical neurones (McKay et al., 2012).

## 4 Discussion

Three main points in regard to antagonism of NMDARs by TCN 201 are made in this study. Firstly, TCN 201 is a selective GluN1/GluN2A NMDAR antagonist showing little antagonism at GluN1/GluN2B NMDARs. Secondly, TCN 201 acts in a non-competitive manner with the extent of its block being dependent on the concentration of the GluN1-site agonist (glycine or d-serine) present in the external salt solution. Thirdly, TCN 201 antagonism of NMDAR-mediated currents shows a negative correlation with their ifenprodil sensitivity and therefore, in combination with ifenprodil, can be used to monitor, pharmacologically, the expression levels of GluN2A and GluN2B NMDAR subunits in neuronal populations.

### 4.1 GluN1-site agonist-dependency of TCN 201 antagonism

TCN 201 antagonism of GluN1/GluN2A NMDAR-mediated currents is glycine (d-serine) dependent. In this respect TCN 201 antagonism resembles that produced by the chemically-related compound TCN 213 (Bettini et al., 2010; McKay et al., 2012). When compared with our recent study of TCN 213 antagonism of NMDARs, the data presented here show that TCN 201 is a more potent antagonist (as was predicted in binding assays performed by (Bettini et al., 2010)). Furthermore, our data also suggest that TCN 201 is unable to block completely NMDAR-mediated responses under conditions where the GluN1-site agonist is present at concentrations that may reflect those found in cerebro-spinal fluid *in vivo* (Fig. 2b, d). The data obtained were fitted with curves whose minima were unconstrained predicted a maximal inhibition, in the presence of saturating glycine (or d-serine), of around 40%. Due to the limited solubility of TCN 201 in our solutions we were unable to verify the precise level of maximum inhibition experimentally but the predicted incomplete block of NMDARs when glycine (or d-serine) is present at ≥30 μM needs to be borne in mind when using this novel GluN2A-selective antagonist.

### 4.2 TCN 201 antagonism is not competitive

Quantitative analysis of antagonist action is best carried out using Schild analysis (Arunlakshana and Schild, 1959) to determine the nature of the antagonism and (if appropriate) the *K*_B_ of the antagonist. Our analysis reveals that TCN 201 does not act in a competitive manner at GluN1/GluN2A NMDARs since the Schild plots for TCN 201 when either glycine or d-serine was used as the GluN1-site agonist deviated from linearity at the highest antagonist concentration used in this study (10 μM). While we were able to fit a line with unity slope to the first three data points of the TCN 201 Schild plots (Fig. 3e, f) the intercept on the *abscissa* perhaps more properly describes the concentration of TCN 201 that gives a *dose ratio* of 2 (the negative logarithm of which will equal the pA_2_ for TCN 201) rather than strictly its *K*_B_ value which should be reserved for purely competitive antagonists (Wyllie and Chen, 2007). Comparison of pA_2_ values for TCN 201 and TCN 213 (7.15 *versus* 5.69) shows that TCN 201 is approximately 30-times more potent at GluN1/GluN2A NMDARs.

The hyperbolic nature of the TCN 201 Schild plot is indicative of an allosteric non-competitive antagonism and the data points were better fitted by an alternative equation that has previously been associated with the description of action of agonist-surmountable non-competitive inhibitors acting at G-protein coupled receptors (Christopoulos and Kenakin, 2002). In the case of these surmountable non-competitive inhibitors, deviations away from unity at higher concentrations of antagonist are due to the saturable nature of the antagonism. Since saturable behaviour can be achieved, allosteric antagonists will shift the agonist concentration-response curve to the right, in a manner similar to competitive antagonism, but according to a defined limit (Christopoulos and Kenakin, 2002). Our estimates of an allosteric *K*_B_^#^ of 56 nM (66 nM) and an allosteric constant of 0.0123 (0.0106), when glycine (d-serine) is used as the GluN1-site agonist, are in excellent agreement with a recent study that has employed a similar analysis of TCN 201 antagonism at recombinant NMDARs (Hansen et al., 2012). In their study, Hansen et al. (2012) propose that TCN 201 binds to a site located at the dimer interface between the GluN1 and GluN2 agonist binding domains which leads to an acceleration of glycine (d-serine) unbinding from the GluN1 subunit. The data we present here are entirely consistent with such a mechanism of action.

Although the allosteric constant is close to zero for TCN 201 (when either glycine or d-serine is used as the GluN1-site agonist) and therefore the Schild plot only begins to deviate from unity at antagonist concentrations above about 3 μM this places a limit on the maximum shift in the dose ratio that can be achieved of about 80–90-fold (=1/*α*). Our previous study with TCN 213 (McKay et al., 2012) generated a Schild plot with a unity slope suggesting a competitive form of antagonism for this compound. It should, however, be noted that relative to its *K*_B_ (2 μM) the highest antagonist concentration used (30 μM) would not have allowed us to observe a deviation from a unity slope if TCN 213 possesses a similar allosteric constant to that of TCN 201.

### 4.3 Utility of TCN 201 in identifying neuronal NMDAR subtypes

There are relatively few antagonists that block GluN2A-containing NMDARs in a manner that allows them to be used to identify unequivocally the subunit composition of native NMDARs (Ogden and Traynelis, 2011). The *K*_B_ values for NVP-AAM077 (Auberson et al., 2002) acting at GluN1/GluN2A and GluN1/GluN2B NMDARs are only different by a factor of 5 (Frizelle et al., 2006) therefore limiting the use of this antagonist. TCN 213 (Bettini et al., 2010) although possessing lower potency than NVP-AAM077 at GluN1/GluN2A exhibits little activity at either recombinant or native GluN2B-containing NMDARs (McKay et al., 2012) allowing it to be used to distinguish, pharmacologically, responses mediated by these two NMDAR subtypes. Our data (Fig. 4) shows that TCN 201, like TCN 213, blocks NMDAR-mediated currents in cortical neurones in a manner that is negatively correlated with the extent of block produced by the GluN2B NMDAR antagonist, ifenprodil (McKay et al., 2012). In primary cultures of ‘young’ neurones, TCN 201 produced little block of NMDA-evoked currents consistent with the notion that such populations are predominantly GluN2B-containing NMDARs (Carmignoto and Vicini, 1992; Crair and Malenka, 1995; Flint et al., 1997; Hestrin, 1992; Sheng et al., 1994; Stocca and Vicini, 1998). Overexpression of GluN2A NMDAR subunits in such cultures gave rise to responses that showed increased sensitivity to TCN 201. Indeed in those cells which showed the greatest block by TCN 201 (and least sensitivity to ifenprodil) the extent of this block indicated that the ifenprodil-insensitive current component was mediated by populations of NMDARs mainly comprised of *only* GluN1 and GluN2A subunits since the amount of block obtained was similar to that seen when TCN 201 antagonized NMDA-evoked responses in oocytes expressing GluN1/GluN2A NMDARs. Finally in older cultures the extent of TCN 201 block was variable but always (negatively) correlated with that of ifenprodil and indicated an increased expression of GluN2A-containing NMDARs, as is to be expected with this stage of development. What remains to be determined is the relative contribution of heterodimeric (GluN1/GluN2A) and heterotrimeric (GluN1/GluN2A/GluN2B) NMDAR combinations in this population. Related to this is the fact that we do not know the potency of TCN 201 when it acts at GluN2A/B-containing heterotrimeric NMDARs – a combination thought to represent a substantial proportion of NMDARs in the adult forebrain (Chazot and Stephenson, 1997; Rauner and Kohr, 2011).

## 5 Conclusion

TCN 201 is a potent selective inhibitor of GluN1/GluN2A NMDARs that displays only minimal activity at GluN1/GluN2B NMDARs. The potency of its antagonism is dependent on the concentration of the GluN1-site agonist and not that of the GluN2-site agonist. Schild analysis demonstrates that the nature of its antagonism is not competitive but rather is consistent with the notion that TCN 201 acts by an allosteric non-competitive mechanism. TCN 201, like TCN 213 (McKay et al., 2012), can be used to monitor, pharmacologically, the change in GluN2 NMDAR subunit expression levels in developing central neurones. Nevertheless, while TCN 201 offers the opportunity to block selectively GluN2A-containing NMDARs care must be taken in experimental designs to take account of (1) TCN 201 block is strongly-dependent on the GluN1-site agonist concentration and (2) TCN 201 possesses a comparatively low solubility in salt solutions that are commonly used when assessing NMDAR function.

## Conflict of interest

The authors state no conflict of interest.
